# Association between hibernating myocardium and collateral circulation in patients with coronary chronic total occlusion

**DOI:** 10.3389/fcvm.2024.1366316

**Published:** 2024-08-02

**Authors:** Yaqi Liu, Yongjun Chen, Feifei Zhang, Bao Liu, Jianfeng Wang, Mei Xu, Yuetao Wang, Xiaoliang Shao

**Affiliations:** ^1^ Department of Nuclear Medicine, The Third Affiliated Hospital of Soochow University, Changzhou, China; ^2^ Clinical Translational Institute for Nuclear Medicine and Molecular Imaging, Soochow University, Changzhou, China; ^3^ Department of Cardiology, The Third Affiliated Hospital of Soochow University, Changzhou, China

**Keywords:** coronary artery disease, chronic total occlusion, collateral circulation, hibernating myocardium, association

## Abstract

**Objective:**

To explore the association between the quantity of hibernating myocardium (HM) and collateral circulation in patients with coronary chronic total occlusion (CTO).

**Materials and methods:**

88 CTO patients were retrospectively analyzed who underwent evaluation for HM using both ^99m^Tc-sestamibi Single photon emission computed tomography (^99m^Tc-MIBI SPECT) myocardial perfusion imaging (MPI) combined with ^18^F-fluorodeoxyglucose positron emission tomography (^18^F-FDG PET) myocardial metabolism imaging (MMI). They were divided into two groups according Rentrop grading: the poorly/well-developed collateral circulation group (PD/WD group, Rentrop grades 0–1/2–3). After adjusting for the potential confounding factors and conducting a stratified analysis, we explored the association between the HM index within CTO region and the grading of collateral circulation.

**Results:**

In the WD group, the HM index was notably higher than PD group (46.2 ± 15.7% vs. 20.9 ± 16.7%, *P *< 0.001). When dividing the HM index into tertiles and after adjusting for potential confounders, we observed that the proportion of patients with WD rose as the HM index increased (OR: 1.322, 95% CI: 0.893–1.750, *P *< 0.001), the proportion of patients with WD was 17.4%, 63.3%, and 88.6% for Tertile 1 to Tertile 3.This increasing trend was statistically significant (OR: 1.369, 95% CI: 0.873–1.864, *P *< 0.001), especially between Tertile 3 vs. Tertile 1 (OR: 4.330, 95% CI: 1.459–12.850, *P *= 0.008). Curve fitting displaying an almost linear positive correlation between the two.

**Conclusion:**

The HM index within CTO region is an independent correlation factor for the grading of coronary collateral circulation. A greater HM index corresponded to an increased likelihood of WD.

## 1 Introduction

Coronary chronic total occlusion (CTO) is defined as a complete blockage of the coronary artery lumen due to atherosclerosis, persisting for over 3 months. Among patients diagnosed with coronary artery disease (CAD) via coronary angiography, the prevalence of CTO is estimated to range between 16% and 50% (1). Successful revascularization of CTO can effectively alleviate angina symptoms, improve the quality of life and cardiac function, reduce the incidence of cardiovascular and cerebrovascular events, and potentially improve prognosis (1–4). However, revascularization of CTO, whether through percutaneous coronary intervention (PCI) or coronary artery bypass grafting (CABG), presents significant technical challenges and is associated with increased post-operative complication risks (5–7). Therefore, a thorough benefit-risk assessment before CTO revascularization is especially crucial (8).

Myocardial hibernation denotes a protective state of the myocardium, which manifests in response to chronic ischemia and hypoxia encountered during coronary chronic total occlusion. This functionally impaired hibernating myocardium has the potential to regain its contractile function following revascularization (9). Collateral circulation within the coronary artery emerges as a response to severe stenosis or occlusion. Retrograde intervention through the collateral coronary circulation has become one of the most important strategies for opening occluded vessels in the treatment of patients with CTO (10, 11), and one of the most important factors in predicting perioperative risk as well as prognosis in patients with CTO (12). Previous studies have shown that Well-developed collateral circulation can effectively preserve hibernating myocardium(HM), improve myocardial ischemia symptoms, and protect left heart function (13–15). According to *post hoc* analyses and observational studies, accurate characterization and quantification of hibernating myocardium can help clarify the benefit-risk ratio and assist in the development of therapeutic strategies (16). Patients with CTO represent a heterogeneous group (8), often presenting with multiple cardiovascular risk factors, such as diabetes mellitus, hypertension, hyperlipidemia, heart failure, and peripheral artery disease (1). Among them, 23% of CTO patients have at least two or more occlusive lesions, and 40% have a history of MI. These patients often exhibit varying degrees of systolic dysfunction, ischemia or hibernating myocardium, scarred myocardium, and collateral circulation. The complex clinical characteristics, coronary circulatory dynamics, and myocardial pathophysiological changes in CTO patients pose challenges and difficulties in studying the association between HM and coronary collateral circulation. Wang et al. (17) and Leite et al. (18) found no significant correlation between the amount of HM and coronary collateral circulation in CTO patients. In addition, Schumacher et al. (19) found that hibernating myocardium was more common in CTO patients with poor collateral circulation, which may be due to a larger area of myocardial perfusion defects and more dysfunctional myocardial segments in patients with poor collateral circulation. There is still controversy about the relationship between HM and collateral circulation, and this inconsistency may arise from the presence of unadjusted confounding factors, including prior myocardial infarction (MI), cardiac function, and myocardial blood flow perfusion. These factors can potentially cloud the association between HM and coronary collateral circulation in different studies. So we introduced the parameter of HM index, aimed to demonstrate whether there is an association between HM index and collateral circulation based on adequate adjustment for confounders.

## 2 Methods

### 2.1 Study population

A retrospective analysis was conducted on patients with CTO who underwent ^99m^Tc-sestamibi single photon emission computed tomography (^99m^Tc-MIBI SPECT) myocardial perfusion imaging (MPI) combined with ^18^F-fluorodeoxyglucose positron emission tomography (^18^F-FDG PET) myocardial metabolism imaging (MMI) for the evaluation of HM in the Department of Nuclear Medicine of the Third Affiliated Hospital of Soochow University from September 2012 to June 2023. The exclusion criteria were as follows: (1) acute coronary syndrome caused by acute coronary occlusion within the last 3 months; (2) significant arrhythmia; (3) dilated cardiomyopathy or hypertrophic cardiomyopathy; (4) serious valvular disease; (5) prior CABG surgery or PCI performed on the occluded vessel; (6) poor image quality preventing the quantification of HM. In total, 88 patients were included in the study.

**Figure 1:**
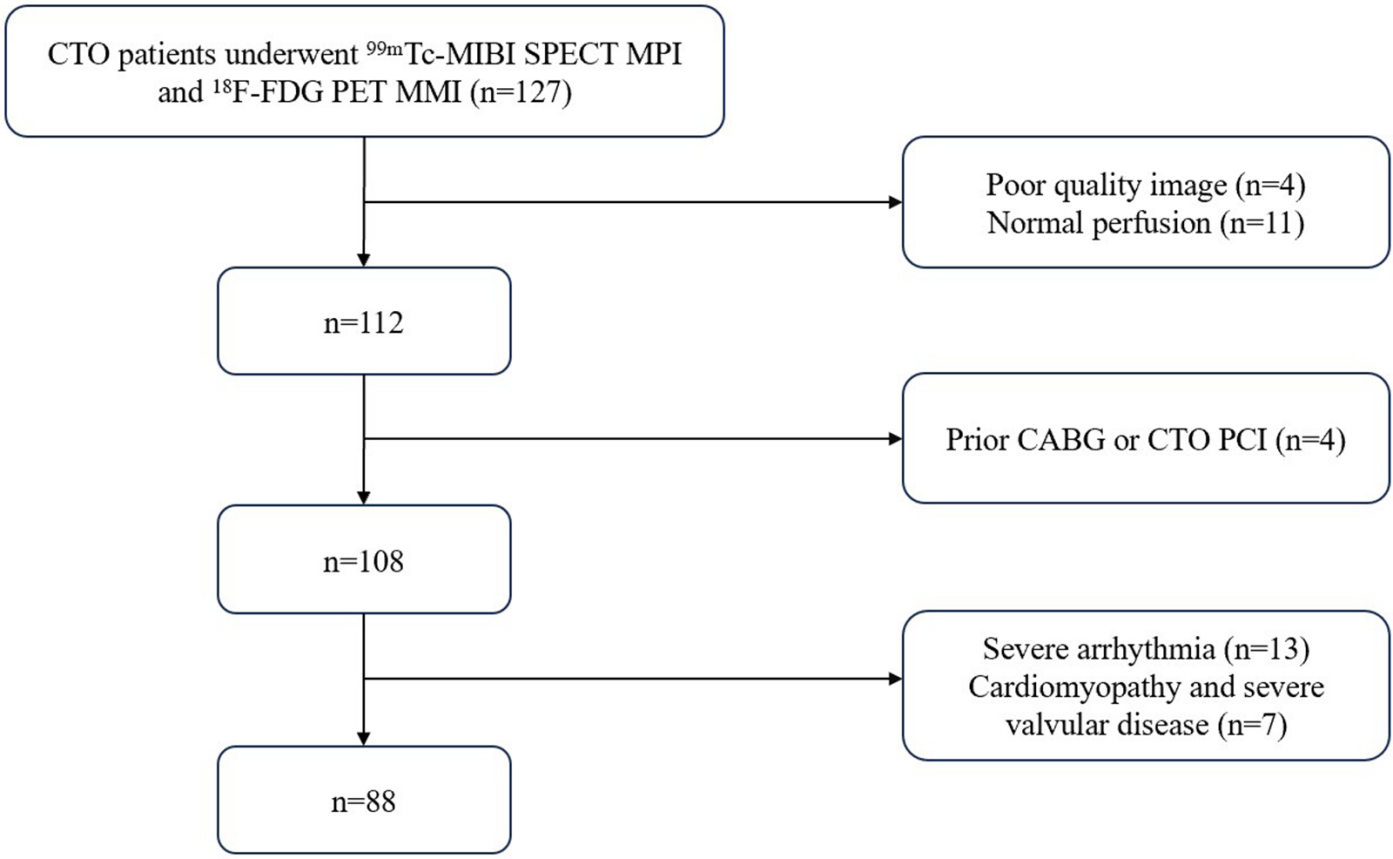
Flow chart of researcher selection.

Baseline characteristics were recorded including body mass index (BMI), hypertension, hyperlipidemia, diabetes, chronic obstructive pulmonary disease (COPD), smoking history, angina with its Canadian class classification of angina pectoris (CCS), New York Heart Association classification of heart failure (NYHA), prior MI and PCI, pathological Q -waves on electrocardiogram (ECG), and echocardiographic parameters including left ventricular end-diastolic dimension (LVEDD), left ventricular end-systolic dimension (LVESD), and left ventricular ejection fraction (LVEF). The protocol of this study adhered to the tenets of the Declaration of Helsinki, was approved by the Ethics committee of our hospital. Flow chart of researcher selection is provided in Figure 1.

### 2.2 ^99m^Tc-MIBI SPECT MPI

Resting gated SPECT MPI was performed 60 to 90 min after injection of ^99m^Tc-MIBI (370 to 555 MBq, radiopurity >95%, procured from Shanghai Xinke Company) in all subjects, using a 2-detector 90° camera (Symbia T16, Siemens Medical Systems, Erlangen, Germany) equipped with a low-energy and high-resolution parallel hole collimator centered on the 140 keV photopeak with a 20% symmetric energy window. An ECG R-wave detector provided gating to acquire 8 emission frames per cardiac cycle. Thirty-two images covering 180° from 45° right anterior oblique view to 45° left posterior oblique view, were acquired with a 128 × 128 matrices and 1.45 magnification. Transaxial images were reconstructed using the filtered back projection method (Butterworth filter; filter function, 0.45; order, 5). Horizontal long-axis, vertical long-axis, and short-axis images were obtained.

### 2.3 ^18^F-FDG PET MMI

The imaging was performed using the Siemens Biograph mCT-s (64) PET/CT system (Germany). Blood glucose levels were managed through a method that encompassed fasting, administering oral glucose, and subsequent intravenous insulin injection. The dosages for both oral glucose and intravenous insulin were determined based on the ASNC PET imaging guideline (20). After adjusting the blood glucose levels, subjects received an intravenous administration of ^18^F-FDG (111 to 185 MBq, radiopurity > 95%, procured from Nanjing Jiangyuan Andi Positron Research & Development Co., Ltd). Sixty minutes post-injection, a cardiac PET-gated acquisition was performed, capturing 8 frames for each R-R interval. This utilized a 3D acquisition method, lasting 10 min per bed position. The images collected were then computationally reconstructed (ultraHD-PET algorithm; iterations, 2; subsets 21). Horizontal long-axis, vertical long-axis, and short-axis images were obtained.

### 2.4 Myocardial perfusion and assessment of HM

Myocardial perfusion/metabolic images were independently and quantitatively analyzed by two nuclear medicine physicians with over 5 years of experience using a blinded approach. And total perfusion deficit (TPD) of LV were assessed by an automated cardiac software package (QPS 2009, Cedars-Sinai Medical Center, Los Angeles, CA, USA). The American Heart Association (AHA) 17-segment scoring method was employed for this analysis. Based on the myocardial region supplied by the CTO vessel, the left ventricular was divided into CTO region and non-CTO region.

Segmental tracer activity was categorized on a 5-point scale to indicate segmental ^99m^Tc-MIBI and ^18^F-FDG uptake: 0 = normal tracer activity; 1 = mildly reduced tracer activity; 2 = moderately reduced tracer activity; 3 = severely reduced tracer activity; and 4 = absence of tracer activity.A scale of 0–5 was used to grade segmental wall motion (0 = normal, 1 = mildly hypokinetic, 2 = moderately hypokinetic, 3 = severely hypokinetic, 4 = akinetic, 5 = dyskinetic). Myocardial segments with perfusion deficit scores ≥2 and wall motion ≥ 1 on at least two consecutive slices in two different axial orientations were defined as segments with reduced perfusion. Myocardial segments with a mismatch in perfusion/metabolism, where the difference in perfusion/metabolism scores was ≥1, were defined as HM segments (21). The number of segments with reduced myocardial perfusion, summed rest score (SRS) in the CTO region, and ^18^F-FDG uptake score in the CTO region was computed (17). Sum of resting perfusion deficit scores for each myocardium segment of CTO region was defined as summed rest score (SRS) in the CTO region. The sum of the ^18^F-FDG uptake scores for each myocardium segment of CTO region was defined as ^18^F-FDG uptake score in the CTO region. The HM index was calculated as follows: (SRS in the CTO region −^18^F-FDG uptake score in the CTO region)/(number of segments with reduced perfusion in the CTO region × 4) × 100% (21).

### 2.5 Coronary angiography

Cardiologists conducted a review of the coronary angiography images, ensuring they were blinded to the patient's clinical data and group allocations. The quality of coronary angiograms and the grading of collateral circulation in the enrolled population were performed with the participation of a cardiologist and a nuclear medicine physician, thus ensuring the accuracy of the data. CTO-ARC proposes that CTOs should be classified as definite and probable CTOs. Definite CTO defined as a luminal occlusion in a coronary artery for an estimated time of ≥3 months with no contrast penetration through the lesion [Thrombolysis In Myocardial Infarction (TIMI) flow grade 0] (22). The collateral circulation of the coronary artery was classified based on the Rentrop grading system (23). According to the Rentrop grades, Grade 0 = no visible collateral filling, Grade 1 = filling of side branches, Grade 2 = partial filling of the epicardial segment of the occluded vessel, and Grade 3 = complete filling of the epicardial segment. Rentrop grades 2–3 were defined as WD, whereas Rentrop grades 0–1 were characterized as PD.

### 2.6 Statistical analysis

Statistical analyses were conducted using R software (Version 4.2.0, http://www.R-project.org). Data following normal distribution were described as mean ± SD. Non-normally distributed data were described as median P50 (P25, P75), and categorical variables were described as counts or percentages. Differences between groups were analyzed using the chi-square test or Fisher's exact test (for categorical variables), one-way ANOVA (for normally distributed continuous variables), and the Kruskal-Wallis test (for skewed continuous variables). The consistency of measurements, both within and between observers, was assessed using the intraclass correlation coefficient (ICC).

Univariate logistic regression was employed to calculate the relationships between various clinical characteristic, examination parameters, and the grading of collateral circulation. A multivariable logistic regression model was established to evaluate the association between the HM index and the grading of collateral circulation, resulting in three models: (1) a univariate model using the HM index as a predictor, (2) multivariate Model I, adjusted for age, gender, (3) multivariate Model II (confounder-adjusted model): assessing the confounding effect of covariates by examining the relationship between the HM index and the grading of collateral circulation before and after introducing the covariates. Variables significantly associated with WD (*P* < 0.10) were considered potential confounders and included in the model. The model provided odds ratios (ORs) and their 95% CIs. Further screening for confounders, confounding factor selection principles: the influence of covariates on the regression coefficient of WD <0.1, and the effect of introducing covariates to the HM index or eliminating covariates from the complete model >10%. We employed the generalized additive model, which is instrumental in identifying non-linear relationships and evaluating potential threshold effects. Alongside this, smooth curve fitting was used to assess the association between the HM index and the grading of collateral circulation. Furthermore, interaction tests and stratified analyses were carried out based on LVEF groupings. *P*-value < 0.05 was indicated statistical significance.

## 3 Results

### 3.1 Comparison of clinical and coronary angiography characteristics and other parameters across different HM index tertiles

A total of 88 patients were included in the study. Their clinical and coronary angiography characteristics, cardiac function and nuclear imaging parameters are presented in Table 1. The HM index was 36.4 ± 20.2%. The intra-observer and inter-observer consistencies were 0.934 and 0.820, respectively, both with *P*-values < 0.001. Based on the HM index, patients were divided into three tertiles: Tertile 1 (0–21.4%), Tertile 2 (25.0–46.9%), and Tertile 3 (50.0–75.0%). Comparisons between these groups are also detailed in Table 1. There were statistically significant differences among the three groups in terms of age, CTO location, the grading of collateral circulation, LVEDD, LVESD, LVEF, TPD, and SRS in the CTO region (all *P*-values < 0.05). However, there were no notable statistical differences for the remaining parameters among the groups.

**Table 1:** Comparison of clinical and coronary angiography characteristics and other parameters across different HM index tertiles.

Characteristics	Total	Tertile 1 (*n* = 23)	Tertile 2 (*n* = 30)	Tertile 3 (*n* = 35)	*P*-value
Age, year	61.0 ± 9.8	59.0 ± 11.1	58.8 ± 8.7	64.1 ± 9.3	0.049*
Gender, *n*					0.294
Female	12 (13.6%)	3 (13.0%)	2 (6.7%)	7 (20.0%)	
Male	76 (86.4%)	20 (87.0%)	28 (93.7%)	28 (80.0%)	
BMI, kg/m^2^	24.9 ± 3.0	25.7 ± 3.8	24.3 ± 2.5	24.8 ± 2.8	0.226
Hypertension, *n*	74 (84.1%)	19 (82.6%)	24 (80.0%)	31 (88.6%)	0.626
Hyperlipidemia, *n*	12 (13.6%)	2 (8.7%)	7 (23.3%)	3 (8.6%)	0.162
Diabetes, *n*	34 (38.6%)	10 (43.5%)	11 (36.7%)	13 (37.1%)	0.857
COPD, *n*	4 (4.5%)	1 (4.3%)	2 (6.7%)	1 (2.9%)	0.762
Smokers, *n*	44 (50.0%)	11 (47.8%)	17 (56.7%)	16 (45.7%)	0.659
Angina, *n*	78 (88.6%)	21 (91.3%)	28 (93.3%)	29 (82.9%)	0.372
CCS class III-IV, *n*	54 (61.4%)	17 (73.9%)	19 (63.3%)	18 (51.4%)	0.219
NYHA class III–IV, *n*	37 (42.0%)	14 (60.9%)	13 (43.3%)	10 (28.6%)	0.051
Prior MI, *n*	53 (60.2%)	17 (73.9%)	16 (53.3%)	20 (57.1%)	0.282
Prior PCI, *n*	21 (23.9%)	6 (26.1%)	6 (20.0%)	9 (25.7%)	0.829
Pathological Q-waves on ECG, *n*	51 (58.0%)	16 (69.6%)	18 (60.0%)	17 (48.6%)	0.274
Coronary angiography					
Coronary dominant, *n*					0.544
Right dominant	74 (84.1%)	18 (78.3%)	27 (90.0%)	29 (82.9%)	
Left dominant	5 (5.7%)	2 (8.7%)	0 (0.0%)	3 (8.6%)	
Codominant	9 (10.2%)	3 (13.0%)	3 (10.0%)	3 (8.6%)	
CTO number, *n*					0.617
Single CTO vessel	67 (76.1%)	18 (78.3%)	21 (70.0%)	28 (80.0%)	
Double CTO vessel	21 (23.9%)	5 (21.7%)	9 (30.0%)	7 (20.0%)	
CTO locations, *n*					
LAD CTO	44 (50.0%)	13 (56.5%)	20 (66.7%)	11 (31.4%)	0.014*
LCx CTO	22 (25.0%)	8 (34.8%)	6 (20.0%)	8 (22.9%)	0.436
RCA CTO	43 (48.9%)	7 (30.4%)	13 (43.3%)	23 (65.7%)	0.024*
Coronary stenosis, *n* (LAD/ LCx/ RCA)					
100%	109 (41.3%)	28 (40.6%)	39 (43.3%)	42 (40.0%)	0.617
70%–99%	92 (34.8%)	20 (29.0%)	31 (34.4%)	41 (39.0%)	0.462
50%–70%	19 (7.2%)	5 (7.2%)	6 (6.7%)	8 (7.6%)	0.962
<50%	44 (16.7%)	17 (24.6%)	13 (14.4%)	14 (13.3%)	0.300
LM ≥ 50%, n	16 (18.2%)	4 (17.4%)	2 (6.7%)	10 (28.6%)	0.073
WD, *n*	54 (61.4%)	4 (17.4%)	19 (63.3%)	31 (88.6%)	<0.001*
Cardiac function and nuclear imaging parameters					
LVEDD (mm)	57.4 ± 8.8	63.8 ± 10.1	57.8 ± 7.9	53.1 ± 5.9	<0.001*
LVESD (mm)	43.5 ± 10.1	50.4 ± 11.2	43.6 ± 9.8	39.1 ± 6.8	<0.001*
LVEF (%)	46.9 ± 11.8	39.6 ± 11.8	47.8 ± 11.7	51.0 ± 9.9	<0.001*
TPD (%)	24.5 ± 12.1	31.4 ± 12.7	25.3 ± 10.2	19.2 ± 10.8	<0.001*
SRS in the CTO region	12.5 ± 6.4	16.0 ± 6.3	13.1 ± 4.9	9.6 ± 6.3	<0.001*

The results are expressed as mean ± (SD)/N (%).

CTO, chronic total occlusion; HM, hibernating myocardium; BMI, body mass index; COPD, chronic obstructive pulmonary disease; CCS, Canadian class classification of angina pectoris; NYHA, New York Heart Association classification of heart failure; MI, myocardial infarction; PCI, percutaneous coronary intervention; ECG, electrocardiogram; LM, left main; WD, well-developed collateral circulation; LAD, left anterior descending artery; LCx, left circumflex artery; RCA, right coronary artery; LVEDD, left ventricular end-diastolic dimension; LVESD, left ventricular end-systolic dimension; LVEF, left ventricular ejection fraction; TPD, total perfusion deficit; SRS, summed rest score.

*P < 0.05.

**Figure 2:**
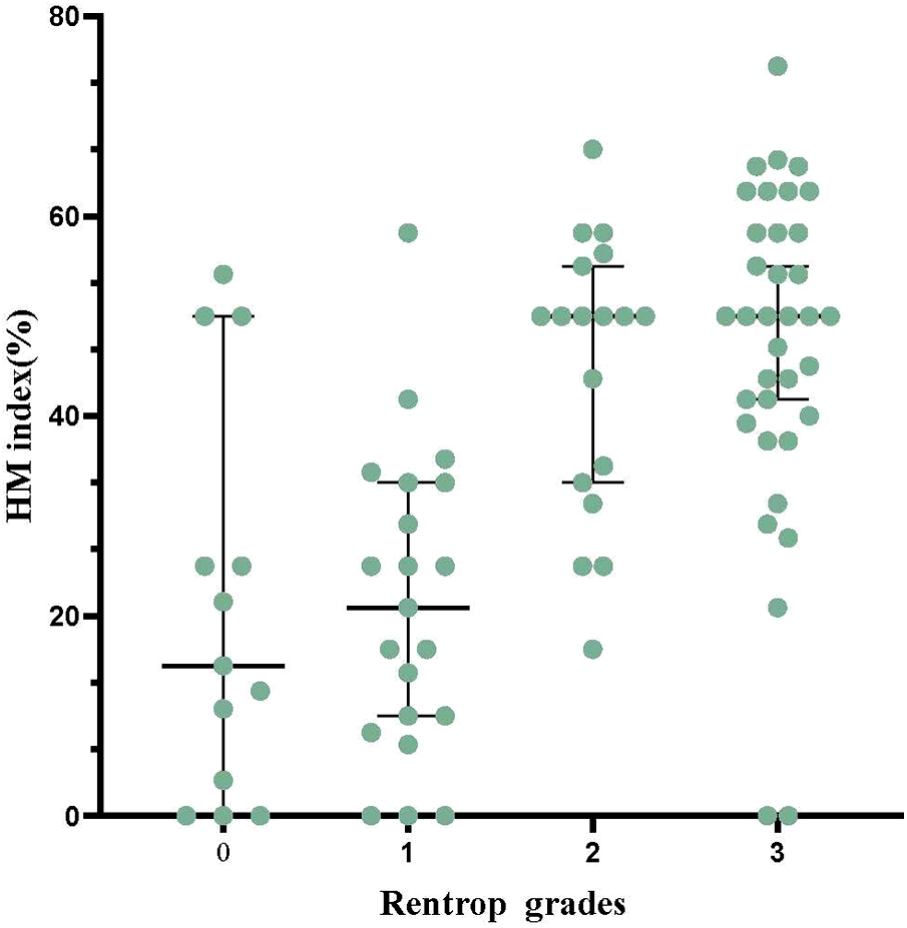
The comparison of HM index between different rentrop grades.

Regarding Rentrop grades, 13 patients (14.8%) were grade 0, 21 patients (23.9%) were grade 1, 18 patients (20.5%) were grade 2, and 36 patients (40.9%) were grade 3. Figure 2 represents the comparison of HM index between different Rentrop grades. Among them, 54 patients (61.4%) had WD, there were 226 segments with reduced perfusion, 195(86.3%) segments with perfusion/metabolism “mismatch” (HM segments), and 31(13.7%) segments with perfusion/metabolism “match”. And 34 patients (38.6%) had PD, there were 166 segments with reduced perfusion, 70(42.2%) segments with perfusion/metabolism “mismatch” (HM segments), and 96(57.8%) segments with perfusion/metabolism “match”. The HM index was significantly higher in patients with WD compared to those with PD (46.2 ± 15.7% vs. 20.9 ± 16.7%, *P *< 0.001). As the HM index increased, moving from Tertile 1 to Tertile 3, there was a corresponding and gradual increase in the proportion of patients with WD: 17.4%, 63.3%, and 88.6%, respectively. The differences among these groups were statistically significant (*P *< 0.001).

### 3.2 Crude association between the clinical and coronary angiography characteristics, other parameters and the grading of collateral circulation

We conducted a univariate logistic regression analysis with the presence of WD, as determined by coronary angiography, serving as the dependent variable (Y = 1). A range of parameters, such as clinical and coronary angiography characteristics, cardiac function, and nuclear imaging parameters, including the HM index, were incorporated as independent variables. The results revealed that angina, prior MI, pathological Q-waves on ECG, left anterior descending artery (LAD) CTO, right coronary artery (RCA) CTO, LVEDD, LVESD, LVEF, TPD, SRS in the CTO region, and the HM index were all potential factors related to the development of WD (all *P* values <0.10). Detailed data can be found in Table 2.

**Table 2:** Crude association between the clinical and coronary angiography characteristics, other parameters and the grading of collateral circulation.

Characteristics	Statistics	OR (95%CI)	*P*-value
Age, year	61.0 ± 9.8	1.027 (0.982, 1.073)	0.246
Age group, year			0.226
<60	42 (47.7%)	1.0	
≥60	46 (52.3%)	1.707 (0.718, 4.057)	
Gender			0.110
Female	12 (13.6%)	1.0	
Male	76 (86.4%)	0.275 (0.056, 1.342)	
BMI, kg/m^2^	24.9 ± 3.0	0.956 (0.827, 1.104)	0.536
BMI group, kg/m^2^			0.388
≤28	76 (86.4%)	1.0	
>28	12 (13.6%)	0.583 (0.172, 1.983)	
Hypertension	74 (84.1%)	2.462 (0.771, 7.861)	0.128
Hyperlipidemia	12 (13.6%)	0.583 (0.172, 1.983)	0.388
Diabetes	34 (38.6%)	0.840 (0.349, 2.023)	0.698
COPD	4 (4.6%)	0.615 (0.083, 4.588)	0.636
Smokers	44 (50.0%)	0.825 (0.350, 1.949)	0.662
Angina	78 (88.6%)	0.152 (0.018, 1.255)	0.080*
CCS class III-IV	54 (61.4%)	0.521 (0.209, 1.297)	0.161
NYHA class III–IV	37 (42.0%)	0.716 (0.301, 1.705)	0.450
Prior MI	53 (60.2%)	0.185 (0.066, 0.518)	0.001*
Prior PCI	21 (23.9%)	0.614 (0.228, 1.655)	0.335
Pathological Q-waves on ECG	51 (58.0%)	0.417 (0.168, 1.036)	0.059*
CTO number			0.568
Singe CTO vessel	67 (76.1%)	1.0	
Double CTO vessel	21 (23.9%)	1.350 (0.482, 3.783)	
CTO locations			
LAD CTO	44 (50.0%)	0.375 (0.154, 0.912)	0.030*
LCx CTO	22 (25.0%)	1.484 (0.534, 4.125)	0.450
RCA CTO	43 (48.9%)	2.471 (1.018, 5.996)	0.045*
LM ≥ 50%	16 (18.2%)	0.565 (0.190, 1.684)	0.306
LVEDD (mm)	57.4 ± 8.8	0.940 (0.893, 0.990)	0.019*
LVESD (mm)	43.5 ± 10.1	0.953 (0.911, 0.996)	0.034*
LVEF (%)	46.9 ± 11.8	1.048 (1.008, 1.090)	0.018*
LVEF group (%)			
<40	22 (25.0%)	1.0	
≥40, <50	22 (25.0%)	2.528 (0.750, 8.522)	0.135
≥50	44 (50.0%)	3.444 (1.183, 10.027)	0.023*
TPD (%)	24.5 ± 12.1	0.929 (0.889, 0.970)	<0.001*
TPD group (%)			0.530
<10	13 (14.8%)	1.0	
≥10	75 (85.2%)	0.667 (0.188, 2.362)	
SRS in the CTO region	12.5 ± 6.4	0.892 (0.827, 0.962)	0.003*
HM index (%)	36.4 ± 20.2	1.087 (1.051, 1.125)	<0.001*

The results are expressed as mean ± (SD)/N (%).

CTO, chronic total occlusion; HM, hibernating myocardium; BMI, body mass index; COPD, chronic obstructive pulmonary disease; CCS, Canadian class classification of angina pectoris; NYHA, New York Heart Association classification of heart failure;MI, myocardial infarction; PCI, percutaneous coronary intervention; ECG, electrocardiogram; LM, left main coronary artery; LAD, left anterior descending artery; LCx, left circumflex artery; RCA, right coronary artery; LVEDD, left ventricular end-diastolic dimension; LVESD, left ventricular end-systolic dimension; LVEF, left ventricular ejection fraction; TPD, total perfusion deficit; SRS, summed rest score.

*P < 0.1.

### 3.3 Multivariate logistic regression for the association between the HM index and the grading of collateral circulation

Both univariate and multivariate logistic regression analyses were performed, considering the continuous variable as well as the tertiles of the HM index. Detailed results can be found in Table 3. For the continuous variable, across the unadjusted (non-adjusted), preliminarily adjusted (Adjust I), and fully adjusted (Adjust II) models, a rise in the HM index was consistently associated with an increased likelihood of observing WD in coronary angiography. The ORs across these models were 1.480, 1.455, and 1.322, respectively, all *P *< 0.001.

**Table 3:** Multivariate regression analysis for the effect of HM index on the grading of collateral circulation.

HM index	Non-adjusted		Adjust I		Adjust II	
	OR (95%CI)	*P*-value	OR (95%CI)	*P*-value	OR (95%CI)	*P*-value
Total	1.480 (1.074, 1.1885)	<0.001	1.455 (1.040, 1.870)	<0.001	1.322 (0.893, 1.750)	<0.001
Tertile 1	1.0		1.0		1.0	
Tertile 2	3.642 (1.239, 10.703)	0.019	3.715 (1.262, 10.935)	0.017	3.209 (1.079, 9.547)	0.048
Tertile 3	5.093 (1.798, 14.426)	0.002	4.982 (1.741, 14.255)	0.002	4.330 (1.459, 12.850)	0.008
*P* for trend	1.559 (1.091, 2.028)	<0.001	1.540 (1.059, 2.022)	<0.001	1.369 (0.873, 1.864)	<0.001

Non-adjusted model adjusts for: None.

Adjust I model adjusts for age, gender.

Adjust II model adjusts for age, gender, prior MI, TPD, and SRS in the CTO region.

Line 1 OR: For the continuous variable HM index, elevated HM index increased the probability of WD in the regression equations for non-adjusted, Adjust I and Adjust II, with ORs of 1.480, 1.455 and 1.322, respectively.

Line 2 &3 OR: For non-adjusted, Adjust I and Adjust II, the proportion of patients with WD in Tertile 2 is 3.642,3.715,3.209 times higher than in tertile 1 (OR = 5.093,4.982,4.330), and in Tertile 3 is 5.093,4.982,4.330 times higher than in tertile 1 (OR = 5.093,4.982,4.330).

Line 3 OR: For the tertile HM index, the trend of elevated HM index increased the probability of WD in the regression equations for non-adjusted, Adjust I and Adjust II, with ORs of 1.559, 1.540 and 1.369, respectively.

CTO, chronic total occlusion; HM, hibernating myocardium; MI, myocardial infarction; TPD, total perfusion deficit; SRS, summed rest score.

When considering the tertiles of HM index, a distinct upward trend was evident across the unadjusted (non-adjusted), preliminarily adjusted (Adjust I), and fully adjusted (Adjust II) regression models. This trend indicated that an increasing HM index significantly associated with the likelihood of having WD. The ORs for these models were 1.559, 1.540, and 1.369, respectively, all *P*-values < 0.001. The non-adjusted model corresponded to the univariate logistic regression analysis. Adjust I accounted for three specific covariates: age, gender. Meanwhile, Adjust II accounted for a broader set of 5 covariates: age, gender, prior MI, TPD, and SRS in the CTO region.

### 3.4 Smooth curve fitting

**Figure 3:**
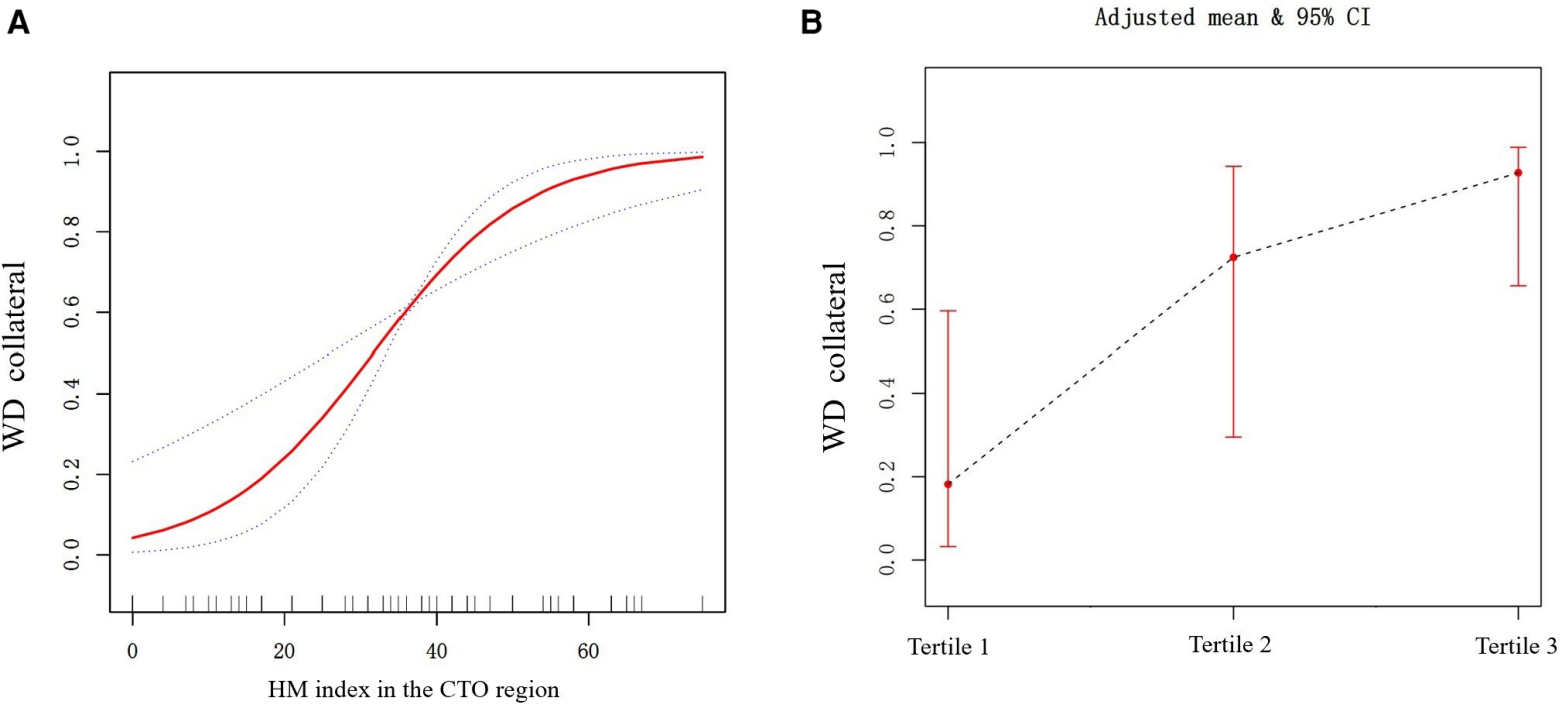
(**A**) The relationship between the HM index and WD (the solid red line indicates the fitted line of the probability of WD and HM index; the blue dotted line is the 95% confidence interval). Adjusted for age, gender, prior MI, TPD, and SRS in the CTO region. (**B**) The relationship between HM index tertiles and WD (the black dotted line indicates the fitted line of the probability of WD and HM index tertiles; the red line is the 95% confidence interval). Adjusted for age, gender, prior MI, TPD, and SRS in the CTO region.

**Figure 4:**
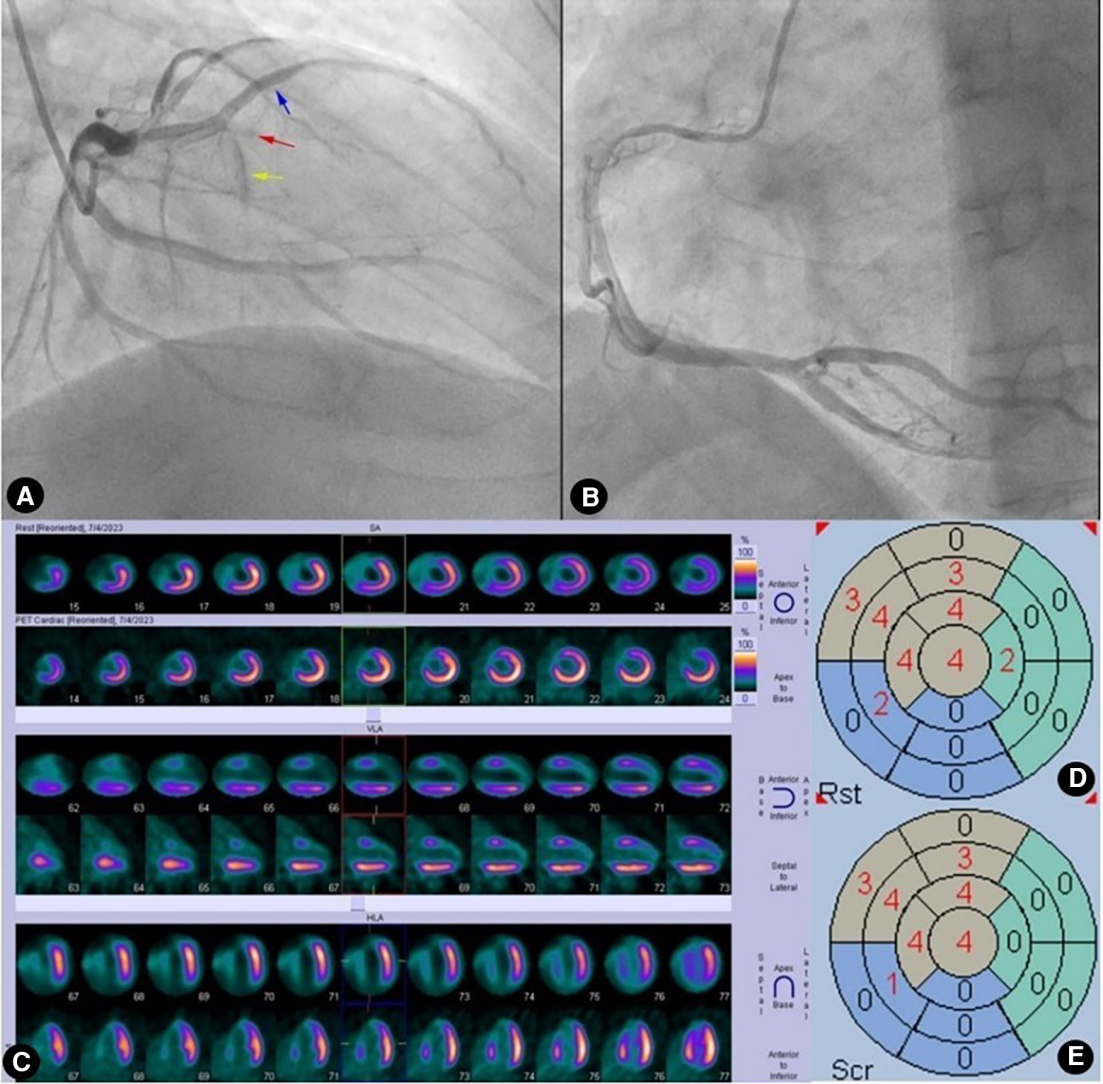
Case 1: male, 60 years old. (**A**) Coronary angiography showed that the left anterior descending artery (LAD) was completely occluded (red arrow) after emitting a diagonal branch (blue arrow) and septal branches (yellow arrow). (**B**) Coronary angiography showed no evidence of collateral circulation. (**C**) Rows 1, 3, and 5 presented ^99m^Tc-MIBI SPECT MPI, demonstrating segmental radiotracer deficits/defects in parts of the anterior wall, septal wall, and apex (six myocardial segments supplied by the LAD). Rows 2, 4, and 6 presented ^18^F-FDG PET MMI. The segments showing radiotracer deficits/defects in the perfusion imaging exhibited no radiotracer uptake in the metabolic imaging, indicating a perfusion/metabolism “match”. (**D**) Scoring for myocardial perfusion imaging. (**E**) Scoring for myocardial metabolism imaging. After calculations, the HM index is (22-22)/(6 × 4) × 100%=0.

**Figure 5:**
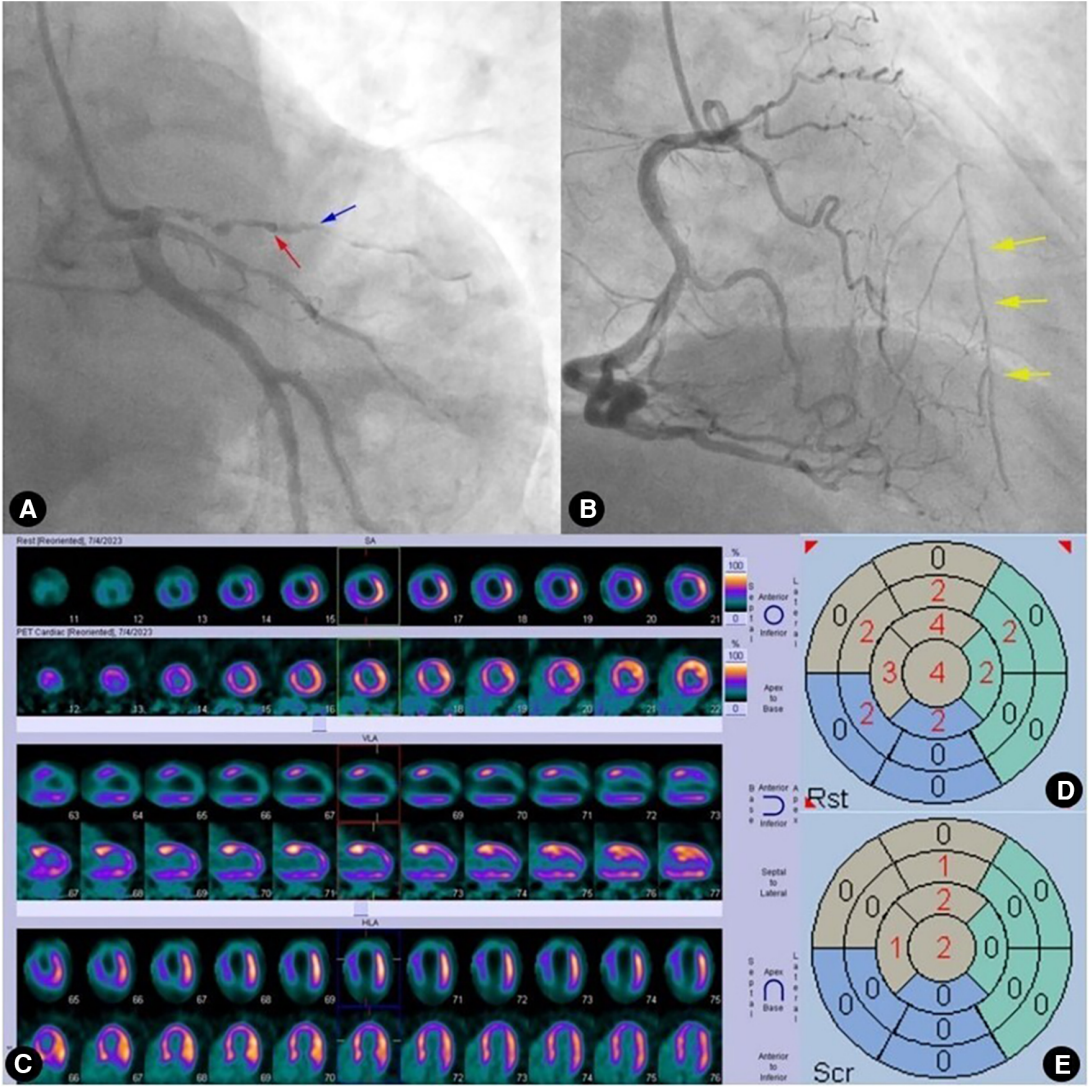
Case 2: male, 59 years old. (**A**) Coronary angiography showed that the left anterior descending artery (LAD) was completely occluded (red arrow) after emitting a diagonal branch (blue arrow). (**B**) Coronary angiography showed WD (Rentrop grade 2) from the right coronary artery (RCA) to the LAD (yellow arrow). (**C**) Rows 1, 3, and 5 presented ^99m^Tc-MIBI SPECT MPI, demonstrating segmental radiotracer deficits/defects in parts of the anterior wall, septal wall, and apex (five myocardial segments supplied by the LAD). Rows 2, 4, and 6 presented ^18^F-FDG PET MMI. The segments showing radiotracer deficits/defects in the perfusion imaging exhibited noticeable radiotracer uptake in the metabolic imaging, indicating a perfusion/metabolism “mismatch”. (**D**) Scoring for myocardial perfusion imaging. (**E**) Scoring for myocardial metabolism imaging. After calculations, the HM index is (15-6)/(5 × 4) × 100% = 45%.

We utilized the generalized additive model to examine the association between the HM index and WD. After fully adjusting for covariates (age, gender, prior MI, TPD, and SRS in the CTO region), it was revealed that a higher HM index was associated with an increased likelihood of having WD. The correlation between the two was approximately linearly positive (degrees of freedom = 1, *P *< 0.001) (Figure 3A). Dividing the HM index into tertiles, the proportion of patients with WD was progressively increased with a rising index. Specifically, the percentages for Tertile 1 to Tertile 3 were 18.2% (95% CI: 3.2–59.6%), 72.4% (95% CI: 29.5–94.3%), and 92.7% (95% CI: 65.6–98.8%), respectively (Figure 3B). Figures 4, 5 present representative cases illustrating the association between the HM index and the grading of collateral circulation.

### 3.5 Stratified analysis

We conducted stratified analyses and interaction tests to evaluate potential factors that might influence the relationship between the HM index and WD. The results indicated that LVEF (both <40% vs. ≥40% and <50% vs. ≥50%) did not significantly alter the association between the HM index and the grading of collateral circulation (*P* for interaction = 0.330) (Table 4).

**Table 4:** Stratified analysis.

Y: WD	*N*	OR	95% CI	*P*-value	*P* (interaction)
LVEF group					0.330
<40%	22	1.100	0.990–1.222	0.077	
≥40%, <50%	22	1.064	0.969–1.169	0.194	
≥50%	44	1.029	0.990–1.068	0.143	
Total	88	1.046	1.013–1.080	0.006	

Adjust for: age, gender, prior MI, TPD, and SRS in the CTO region.

CTO, chronic total occlusion; MI, myocardial infarction; TPD, total perfusion deficit; SRS, summed rest score; WD, well-developed collateral circulation.

## 4 Discussion

Our study found that patients with WD in the CTO region had a significantly higher HM index compared to those with PD. After thoroughly adjusting for potential confounders and interaction effects and fitting the curve, it was observed that the proportion of patients with WD was progressively increased with a rising HM index. This relationship was approximately linearly positive.

Coronary collateral circulation are interatrial connections that supply blood flow to a vascular territory when its primary coronary artery is obstructed (24, 25). Studies indicate that well-developed collateral arteries are present in approximately 35% of patients with CAD experiencing significant hemodynamic abnormalities (26). The formation of well-developed collateral circulation can not only limit the extent of transmural myocardial infarction, improve symptoms of myocardial ischemia, and maintain myocardial survival (13–15) but also holds significant predictive value for the choice and success rate of revascularization procedures for CTO, especially PCI (10, 11). However, due to the spatial resolution limits of coronary angiography, only channels greater than 100–200 μm in diameter can be visualized, leading to a significant underestimation of coronary collateral circulation in CTO patients (27). Clinical studies have found that the development of coronary collateral circulation in CTO patients is influenced by various factors, such as renal insufficiency, diabetes, myocardial infarction, inflammatory factors, and the site of the CTO lesion, with significant interindividual variability (13, 28, 29). This study revealed that CTO patients with WD exhibited significantly lower instances of angina, myocardial infarction, pathological Q-waves on ECG, left ventricular remodeling, and perfusion deficits in the CTO region compared to those with PD. On the other hand, the LVEF, especially in those with an LVEF greater than 50%, and the HM index, were significantly higher in patients with WD, aligning with previous research findings (13, 30, 31). Thus, several factors influenced the development of coronary collateral circulation in CTO patients, with ischemic/hibernating myocardium due to coronary occlusion being significantly associated with the formation of these collaterals.

Although ^18^F-FDG PET MMI is the most commonly used, highly sensitive, and highly accurate imaging technique for clinically evaluating and quantifying HM (32), it is still susceptible to interference from several factors, such as diabetes, cardiac function, myocardial infarction, and the scope and quantity of necrotic myocardium (30). In light of prior research findings (17, 33), this study incorporated the HM index of the CTO region as comprehensively as possible. This was done to mitigate the confounding effects and interference arising from myocardial infarctions outside of the CTO region. This study found that this indicator had excellent repeatability, with intra-observer and inter-observer consistencies reaching 0.934 and 0.820, respectively (*P *< 0.001 for both). At the same time, this study discovered that the HM index was significantly correlated with patient age, coronary angiography features (CTO location and collateral circulation), left ventricular remodeling, and the scope of myocardial perfusion deficits, which was in line with the study by Shokry KA and others (31). Evidently, when studying the association between the grading of collateral circulation and the HM index, there are many confounding factors (related both to the dependent variable of collateral circulation and the independent variable of the HM index). To the best of our knowledge, we, for the first time, systematically excluded interference from the aforementioned confounding factors, truly elucidating their relationship.

The 2016 ESC guidelines for Heart Failure (HF) classified HF for the first time as heart failure with preserved ejection fraction (HFpEF), heart failure with mid-range ejection fraction (HFmEF) and heart failure with reduced ejection fraction(HFrEF). The clinical phenotypes of HFmEF patients are different from those of other types of heart failure, and the potential therapeutic effects are different. If not controlled in time, these patients are prone to develop HFrEF and aggravate the poor prognosis of HF patients. However, we did not find association by stratified analysis of LVEF between the HM index and the grading of collateral circulation, further studies are needed to determine the effect of LVEF group assignment.

Regarding the ability of collateral circulation in CTO patients to predict HM and its quantity and whether a correlation exists between the two, previous studies have been contentious. Some scholars (8, 17, 18, 34, 35) have found no significant correlation between them. They have surmised that due to the spatial resolution of coronary angiography, underestimated collateral circulation cannot effectively and accurately predict HM and its quantity. The maintenance of myocardial viability and functional recovery are more related to the integrity of the microcirculation than to collateral circulation (36). However, in a study by He et al. (37), when inclusion criteria are limited to patients with single coronary CTO lesions and stratified based on previous myocardial infarction history, they have found that WD preserves myocardial viability. Shokry et al. (31), using CMR, have found that the sensitivity and specificity of good collateral circulation in predicting myocardial viability are 72% and 74%, respectively. They suggest that choosing the appropriate imaging method and parameters is crucial for assessing HM in CTO patients with collateral circulation. This study aligns with the findings of the two aforementioned studies (31, 37), revealing that the higher the HM index, the higher the likelihood of WD. Notably, for Tertile 3 of the HM index, up to 92.7% had WD, suggesting an approximate linear positive correlation. Nevertheless, we also observed that a certain proportion of CTO patients with PD had a higher quantity of HM in the CTO region. This could be related to the pathophysiological mechanisms of HM formation, and collateral circulation isn't the only influencing factor for the preservation of HM (38, 39). We posited that the debate over their relationship primarily arose from the presence of numerous confounding variables, the significant heterogeneity among study groups, and inconsistencies in imaging techniques and criteria used to identify HM.

Patients with chest pain undergoing coronary angiography frequently have multiple coronary artery stenoses as well as occluded vessels in multiple coronary artery supply regions. In clinical practice, when chronic total occlusive lesions and left ventricular wall motion abnormalities are detected on coronary angiography, myocardial activity in the region of the myocardium corresponding to the occluded vessel should be further analyzed. The present study demonstrates a correlation between the presence of well-developed collateral circulation and hibernating myocardium, emphasizing the need for further image analysis. By assessing HM using ^18^F-FDG PET myocardial metabolism imaging and adjusting for the potential confounding variables, we clarified the association between HM in the CTO region and collateral circulation. Therefore, in order for patients to obtain a corresponding improvement in cardiac function and prognosis after revascularization, myocardial activity should be analyzed more thoroughly when coronary angiography shows the presence of collateral circulation.This insight offered crucial guidance for risk stratification, clinical decision-making, and choosing surgical approaches for CTO patients. Consequently, this enabled more personalized treatment strategies, ensuring the best possible outcomes for patients.

With the clinical practice of PET assessment of HM, the development of artificial intelligence-assisted diagnosis and the exploratory application of PET/magnetic resonance (MR) have provided new ideas for PET assessment of HM in recent years (40). AI not only improves the automatic detection and segmentation of raw images, but also facilitates the integration of multiple clinical profiles and imaging data to improve diagnostic and predictive efficacy, and to realize accurate diagnosis and risk stratification of patients (41). Integrated PET/MR is capable of simultaneously evaluating the morphologic structure of the heart, function and metabolism, and has become an important noninvasive screening technique for cardiovascular diseases (42). We look forward to more technical advances and standardized improvements in image interpretation methods to better achieve accurate assessment of HM and provide better guidance for clinical treatment as well as prognostic assessment.

### 4.1 Limitations

This study had the following limitations. Firstly, the study was a retrospective cross-sectional investigation with a relatively small sample size. While potential confounders, such as left main lesions and previous PCI surgeries, were incorporated into the study, these populations were not part of the exclusion criteria. Despite screening for confounders and adequately correcting for them, they are still affected by residual confounding. Further large-scale prospective studies are needed for verification. Secondly, the study included a highly selected population of patients referred for assessment of viability for possible revascularization of the CTO due to ischemic equivalent symptoms (chest pain/dyspnea), which of course increases the likelihood of viable myocardium within CTO territories and exists the possibility of referral bias. In addition, due to the large time span of the data, low hospitalization rates in the earlier period and technical and other issues caused a degree of selection bias. Thirdly, we did not incorporate some figures on CTO characteristics such as ostial location, blunt occlusion stump, angle formation of the occluded segment, proximal fibrous cap of the CTO, length of the occlusion, bending, calcification and so on. Fourthly, the study lacked stress myocardial perfusion imaging, making it impossible to assess the interrelationship between coronary collateral circulation and myocardial ischemia. Finally, the study did not have follow-up information post-revascularization surgery, preventing the assessment of the mutual relationship between coronary collateral circulation, the HM index, and the prognosis of CTO patients.

## 5 Conclusions

In conclusion, this retrospective study utilized non-invasive nuclear imaging to quantitatively evaluate HM and coronary angiography to assess the grading of collateral circulation in CTO patients. After adequately adjusting for confounding and interacting factors and fitting curves, we found that the higher the HM index, the higher the likelihood of WD, with the two showing an approximate linear positive correlation. Understanding this association laid the groundwork for building prognostic prediction models following CTO revascularization and for managing confounding variables.

## Data Availability

The original contributions presented in the study are included in the article, further inquiries can be directed to the corresponding authors.
